# Understanding the Mechanism of Formation of a Response to Juglone for Intact and Immobilized Bacterial Cells as Recognition Elements of Microbial Sensors: Processes Causing the Biosensor Response

**DOI:** 10.3390/bios11020056

**Published:** 2021-02-21

**Authors:** Elena V. Emelyanova, Inna P. Solyanikova

**Affiliations:** 1 Laboratory of Biosensor, G.K. Skryabin Institute of Biochemistry and Physiology of Microorganisms, Pushchino Center for Biological Research of the Russian Academy of Sciences, Pushchino, 142290 Moscow Region, Russia; 2 Laboratory of Microbial Enzymology, G.K. Skryabin Institute of Biochemistry and Physiology of Microorganisms, Pushchino Center for Biological Research of the Russian Academy of Sciences, Pushchino, 142290 Moscow Region, Russia; innas@ibpm.pushchino.ru

**Keywords:** 5-hydroxy-1,4-naphthoquinone, *Rhodococcus* sp. 3, *Pseudomonas* sp. 4 (c4), biosensor approach, amperometry, juglone 3-monooxygenase, reactive oxygen species

## Abstract

Microbial reactor sensors (based on freshly harvested intact microbial cells) or microbial membrane sensors (based on immobilized microbial cells) can be used as convenient instruments for studying processes that cause the response of a biosensor, such as the properties of enzymes or the characteristics of metabolism. However, the mechanisms of the formation of biosensors responses have not yet been fully understood to study only one of these processes. In this work, the results of studies on the formation of a response to juglone for intact and immobilized bacterial cells used as receptors are presented. It was shown that the contribution of reactive oxygen species (ROS) to the formation of the biosensor response depends on the culture receptor and the form of juglone, quinone, or phenolate used. The response to the quinone form of juglone both for intact and immobilized cells of catalase-positive actinobacterium is formed regardless of the presence of ROS. The response of freshly harvested intact actinobacterial cells was caused by the rate of the enzymatic conversion of juglone. The rate of the response of immobilized actinobacterial cells was influenced by the activity of transport systems and metabolism. The response of immobilized pseudomonad cells was caused by the transport of juglone into cells, the inhibitory effect of juglone-induced ROS, and juglone metabolism.

## 1. Introduction

Juglone is a natural compound of the naphthoquinone group, 5-hydroxy-1,4-naphthoquinone, C_10_H_6_O_3_. Juglone was obtained for the first time in 1851 while processing walnut fruits. Today, natural sources of juglone are plants such as black walnut (*Juglans nigra*) and white walnut (*Juglans cinerea*) and microorganisms; juglone is also obtained synthetically [1,2].

**Figure 1:**
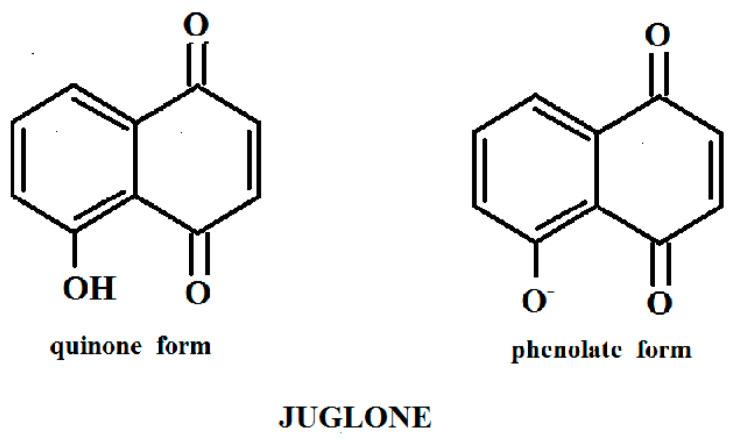
The structural formulae of the quinone and phenolate forms of juglone.

It is known that naphthoquinones are slightly soluble in water. A small amount of juglone (a quinone form) dissolves in water [3]. When juglone is subjected to aqueous NaOH solution, hydrogen is replaced by metal in the hydroxyl group in the aromatic ring (a phenolate form). A phenolate form of juglone becomes water-soluble. The structural formulas of the quinone and phenolate forms of juglone are shown in Figure 1.

Juglone is widely used in industry, agriculture, and medicine. This reddish-yellow crystalline substance is a natural pigment that is used in the textile industry as a natural dye for clothing and fabrics and in the cosmetics industry [4]. Juglone (an allelopathic naphthoquinone) is used as a herbicide that slows down the growth of other plants [5,6,7]. This substance has a relatively wide spectrum of antimicrobial action, which is especially important against pathogenic microorganisms. Thus, it was used in the food industry as a preservative for soft drinks and wines [8,9,10]. The fungicidal and antibacterial properties of juglone are used in medicine for the treatment of fungal and bacterial infections, and for complex therapy to fight many diseases [11,12]. Recently, the cytotoxic effect of juglone has been used against various human tumor cells; this naphthoquinone is used as an agent for stimulating the apoptosis of human leukemia cells [13,14].

It was found that when naphthoquinones penetrate into a cell, they can induce oxidative stress [15] as a result of the formation of reactive oxygen species. Therefore, juglone is used to study the resistance of microorganisms to oxidative stress [16,17,18,19].

The enzyme that initiates the decomposition of juglone under aerobic conditions is juglone 3-monooxygenase (juglone hydroxylase, EC 1.14.99.27) [20,21,22]. The enzyme belongs to the family of oxidoreductases and catalyzes a chemical reaction that occurs with oxygen consumption [23]:

5-hydroxy-1,4-naphthoquinone + AH_2_ + 1/2 O_2_ = 3,5-dihydroxy-1,4- naphthoquinone + A + H_2_O.

A rapid method for the determination of juglone is based on application of sensor analyzers. A microbial sensor can be used for these purposes, for which the response to juglone is evaluated by the change in oxygen consumption by microbial cells in response to substrate addition, since the metabolism of juglone is initiated by an enzyme–substrate interaction that occurs with oxygen consumption. The development of a laboratory model of such a microbial sensor was started at IBPM RAS [24]. Moreover, it was shown that the model of a microbial amperometric sensor for juglone can be employed to study the actinobacterial metabolism of juglone [25]. The responses of microbial amperometric sensors based on intact cells (a microbial reactor sensor) or immobilized cells (a microbial membrane sensor) are caused by different processes [26]. However, the mechanisms of the formation of biosensor responses have not yet been fully understood to study only one of the processes. The effects of juglone on bacterial metabolism using intact freshly harvested and immobilized resting bacterial cells were further explored in this research.

The aim of this research was to study the formation of a response to juglone (5-hydroxy-1,4-naphthoquinone) for intact and immobilized bacterial cells used as receptors for microbial reactor sensors or microbial membrane sensors.

## 2. Materials and Methods 

### 2.1. The Microorganism and Culture Conditions

Non-spore-forming actinobacterial cells of *Rhodococcus* sp. 3 and bacterial cells of *Pseudomonas* sp. 4(c4), stored in a culture collection created by the authors of this study, were used in the present study. The cultures were maintained on the agarized Luria–Bertani (LB) medium at +4 °C and passaged every six months.

### 2.2. Preparation of A Set of Standard Solutions of Juglone and Reagents Used

In this work, the following reagents were used: KH_2_PO_4_ (Panreac, Panreac, Spain Spain); NaOH (Reachem, Russia); CuSO_4_ × 5H_2_O (Reachem, Russia); acetone (Component-reaktiv, Russia); catalase from bovine liver (Sigma-ALDRICH, USA); medium L.B. (Liofilchem, Italy); malt extract agar (Pronadisa, Spain).

Crystalline substance, juglone(5-hydroxy-1,4-naphthoquinone, Aldrich, H47003-1G), was used to prepare a 1 × 10^−1^ M stock solution of juglone in acetone. Using the stock solution, 1 × 10^−2^ M, 1 × 10^−3^ M, and 1 × 10^−4^ M solutions of juglone in acetone were prepared by the method of successive dilution. During the preparation of the dilutions and measurements, the solutions were kept in hermetically sealed vessels without access to light. Similarly, 1 × 10^−1^ M, 1 × 10^−2^ M, 1 × 10^−3^ M, and 1 × 10^−4^ M solutions of juglone were prepared using 5% NaOH solution and distilled water as solvents. The resulting alkaline solutions contained alkali in an amount of 0.008 g/mL.

### 2.3. Formation of Recognition Elements on The Basis of Intact or Immobilized Bacterial Cells

*Rhodococcus* sp. 3 and *Pseudomonas* sp. 4 (c4) were grown on malt agar and on the agarized LB medium, respectively. Bacterial biomass was harvested and suspended in a 50 mM K-Na-phosphate buffer (pH 7.0) to give a final concentration of 100 mg wet weight per mL. Suspension of freshly harvested bacterial cells (intact cells) was immediately used for formation of the microbial reactor sensor [27]. The remaining part of suspension was used to prepare receptor elements (immobilized resting cells) of the microbial membrane sensor. For this purpose, 10 μL of bacterial cell suspension were immobilized by physical adsorption on Whatman paper: the suspension was spotted onto a 4 × 4 mm^2^ piece of paper. The obtained receptor elements were air-dried within 30 minutes at room temperature. Receptor elements were kept in a refrigerator *at +4 °C for 24 h and then used for measurements or stored in the* refrigerator.

### 2.4. Determination of The Response to Juglone for Freshly Harvested Intact Cells (Amodel of The Microbial Reactor Sensor) and Immobilized Resting Cells (A Model of The Microbial Membrane Sensor)

**Figure 2:**
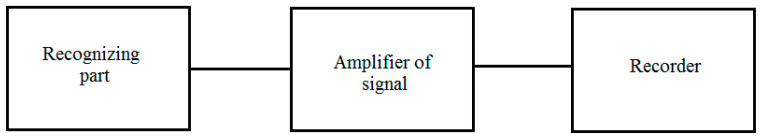
The construction of laboratory models of biosensors.

Laboratory models of microbial sensors were formed with the use of intact or immobilized cells of bacteria. The designs of recognizing parts of the microbial reactor sensor and microbial membrane sensor were given in our article [27]. Figure 2 depicts the construction of laboratory models of biosensors. The obtained laboratory models were employed to detect a response of bacterial cells to juglone (at 20–22 °C). Thus, when the base respiration rate of bacterial cells stabilized, juglone solution was injected into a 5 mL open measuring cell with a magnetic stirrer. The measuring cell contained the air-saturated 50 mM K-Na-phosphate buffer, pH 7.0, (for the microbial membrane sensor) or cell suspension in the same buffer (for the microbial reactor sensor). A change in microbial cells’ respiration after the addition of juglone led to a change in the oxygen level at the working area of an electrode (for the membrane sensor) or to a change in all buffer volume (for the reactor sensor). The change in oxygen concentration was measured by the Clark-type oxygen electrode, which transduced the signal resulting from the interaction of a biological element with juglone into an electrical signal. An amplifier (Ingold 531-04 O_2_ Amplifier, Switzerland-USA) was used to amplify this signal, and a two-coordinate recorder (X-Y Recorder-4103, Czech Republic) was used to record electrical signals. The recorded rate of signal change was proportional to the rate of alteration in oxygen consumption by microbial cells in response to the juglone injected. Based on this, the first derivative of the electrode current change in the response to juglone injection (the rate of response to juglone, pA/s) was calculated. The obtained parameter characterized the rate of change in oxygen consumption by bacterial cells in response to juglone (cells’ response to juglone).

In order to plot the “response-concentration” dependence, sets of standard solutions of juglone in acetone or in NaOH solution with different concentrations of juglone were used. The volume of juglone solution added into the measuring cell was no more than 20 μL.

### 2.5. Statistics

The presented results were averaged values of the measurements which were taken in triplicate in two independent series of experiments. Statistical analysis of the data was carried out using a Student’s *t*-test (*p* < 0.05). It was thought that the differences between the values were statistically significant if the confidence intervals failed to cross.

## 3. Results and Discussion

### 3.1. Response of Intact and Immobilized Cells of Rh. sp. 3 to The Phenolate Form and Quinone Form of Juglone

The response of microbial cells to juglone has been studied in water medium. Only a small amount of the quinone form of juglone dissolves when it is added to water. Bearing this in mind, before the addition of juglone into the measuring cell, it was dissolved in acetone and, after that, in order to minimize the effect of acetone on the cell response, small quantities of acetone solution (the quinone form of juglone) were injected into the aqueous solution. A water-soluble alkaline solution of juglone (the phenolate form) without any treatment was added into the measuring cell. The addition of a small amount of alkali or acetone did not affect the cell respiration (data not shown).

**Figure 3:**
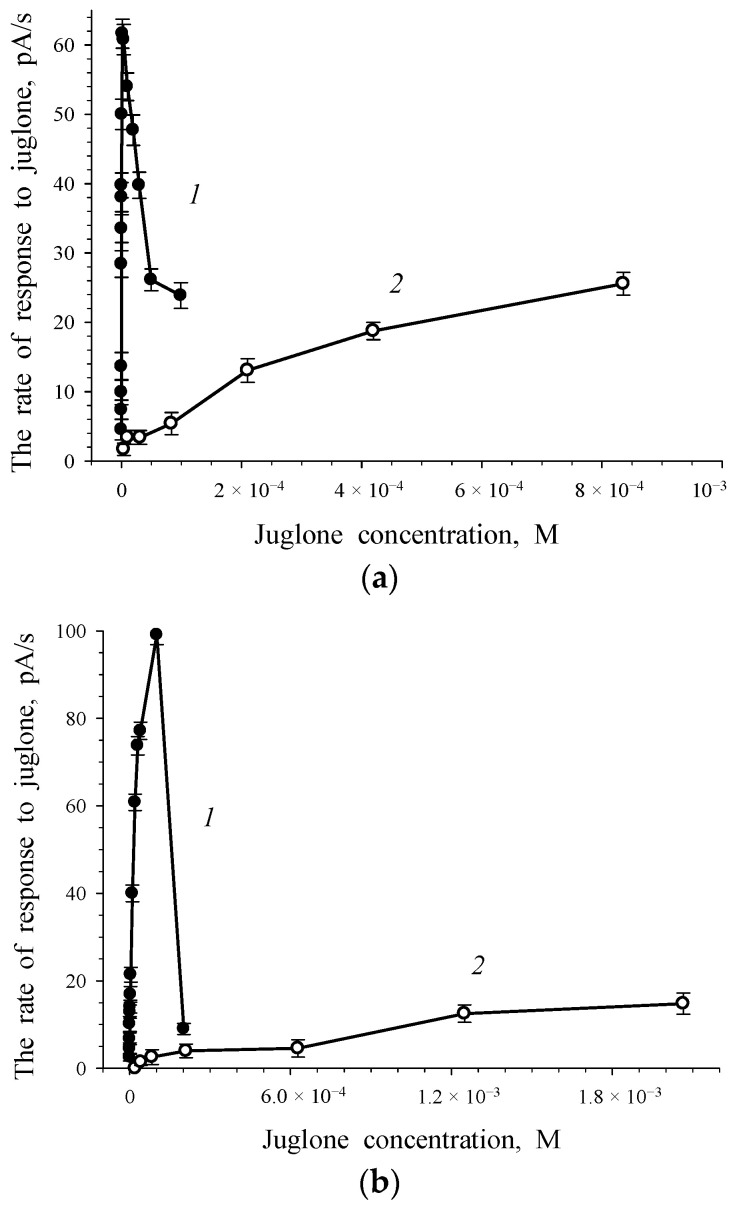
Dependences of the rate of the response to juglone on juglone concentration for intact (**a**) and immobilized (**b**) cells of *Rhodococcus* sp. 3 when the quinone form (curve *1*) or phenolate form (curve *2*) of juglone were used.

The phenolate form and quinone form of juglone were used to plot the curves of the dependence of the rate of the response to juglone on the initial juglone concentration for intact and immobilized cells of *Rhodococcus* sp. 3 (Figure 3). The measurements were made in a buffer at neutral pH (*2.4.* in Materials and Methods). The addition of a water-insoluble substance—for example, acid in the form of water-soluble salt—is the conventional microbiological technique. However, in our case the curves plotted for the phenolate form and quinone form of juglone differed significantly. The rate of response to the quinone form of juglone was an order of magnitude higher than that of the phenolate form, and it attained its maximum when the substrate concentration was an order of magnitude lower. It was recorded both for intact (Figure 3a) and immobilized (Figure 3b) cells of *Rhodococcus* sp. 3. A decrease in the response to the quinone form of juglone (the substrate) was observed when the juglone concentration was above 4×10^−^^6^ M and 1×10^−^^4^ M for intact and immobilized *Rhodococcus* sp. 3 cells, respectively, as a result of substrate inhibition.

The detected difference in responses was obviously due to the presence of a hydroxyl group in the aromatic ring of the quinone form of juglone, which was involved in the induction of oxidative stress. However, there was no phenolic hydroxyl in the phenolate form of juglone. Therefore, both forms of juglone were used in the study of the cells’ response to juglone.

### 3.2. The Effect of Enzyme-Substrate Interaction on The Formation of Responses to Juglone for Intact and Immobilized Rhodococcus sp. 3 Cells

The rate of response to the substrate for intact cells was caused by the rate of enzyme–substrate interaction in the presence of the enzyme, which initiates substrate metabolism. It was shown in our previous studies with the laboratory model of the microbial reaction sensor formed with the use of intact cells of *Rhodococcus opacus* 1CP [28].

Juglone 3-monooxygenase is the enzyme that initiates the metabolism of juglone. We previously reported on the presence of a constitutive enzyme in rhodococci [19]; this was indirectly evidenced by the insensitivity of *Rhodococcus opacus* 1CP to oxidative stress during cultivation in the presence of juglone [18]. “Response-concentration” curves (Figure 3a) were plotted for 3 *Rhodococcus* sp. intact cells grown on the medium in the absence of juglone (without enzyme induction by substrate). There is no inducible enzyme in non-substrate-grown cells. The rate of intact cells’ response to substrate is 0 pA/s or does not exceed 1–1.5 pA/s in the presence of the base concentration of an inducible enzyme in non-substrate-grown cells. It should be noted that in the case of *Rhodococcus* sp. 3 (Figure 3a), the rate of non-juglone-grown cells’ response to the quinone form of juglone was 60 pA/s. Hence, this indicated that juglone 3-monooxygenase of the culture (*Rhodococcus* sp. 3) is a constitutive enzyme.

**Figure 4:**
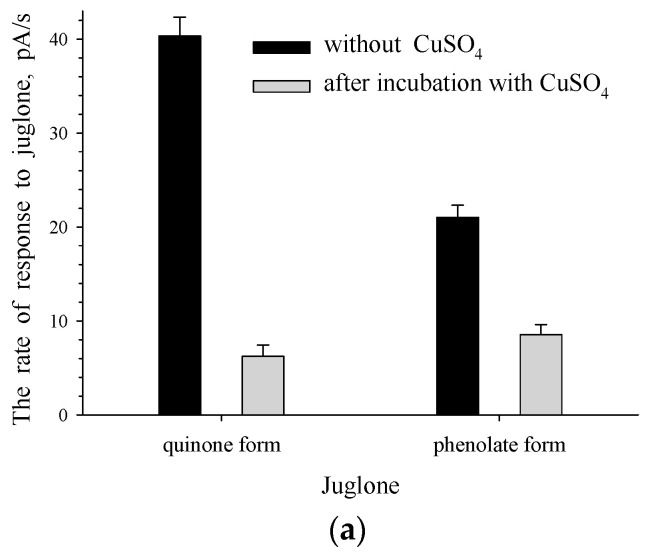

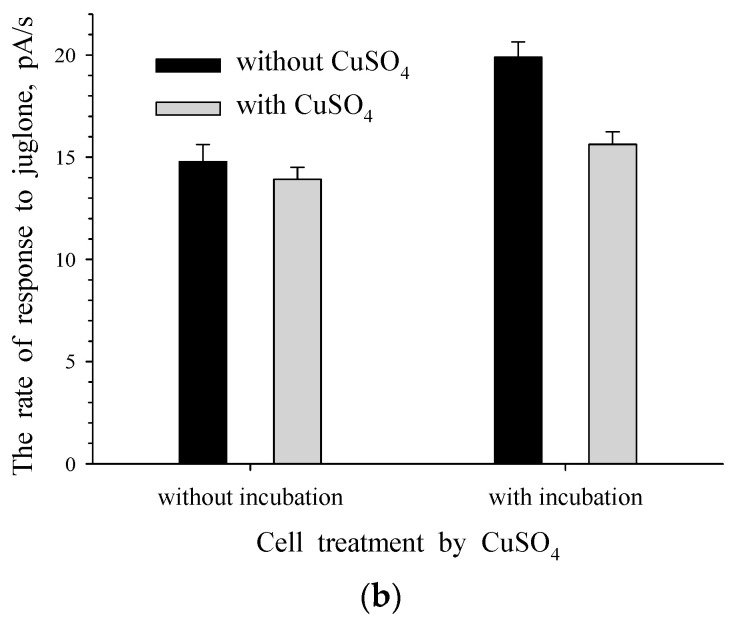
Responses to juglone for intact (**a**) and immobilized (**b**) cells of *Rhodococcus* sp. 3 in the presence and absence of CuSO_4_ (without incubation) and after incubation in the presence and absence of CuSO_4_ (with incubation).

Regarding juglone 3-monooxygenase of *Pseudomonas putida* bacterium, Rettenmaier and Lingens reported [20] that 0.5 mM of CuSO_4_ can inhibit the enzyme activity completely. When copper sulfate inhibits the activity of juglone 3-monooxygenase of *Rhodococcus* sp. 3, the rate of interaction between the enzyme and juglone will be reduced. To estimate a contribution of the enzyme-substrate interaction to the formation of *Rhodococcus* sp. 3 cells response to juglone, intact and immobilized cells were incubated in the presence of CuSO_4_ before the addition of juglone (Figure 4).

The presence of CuSO_4_ led to a significant decrease in the rate of the response to juglone for intact cells—for example, from 40 to 8 pA/s for the quinone form of juglone (Figure 4a). CuSO_4_ inhibits the activity of the enzyme, which initiates the transformation of the substrate and causes the cells’ response to the substrate. Thus, our results demonstrate that the rate of the response to juglone for the microbial reactor sensor based on intact cells of *Rhodococcus* sp. 3 was mainly caused by the rate of enzymatic transformation of juglone.

The decrease in the rate of the response to juglone observed for immobilized cells under the influence of CuSO_4_ was less significant (Figure 4b): the observed rate reduction was only 22%. It should be noted that the incubation time in the presence of CuSO_4_ was not optimized. It is known [26] that in the case of a microbial membrane sensor formed on the basis of immobilized cells, the response to a substrate is caused by several processes. When juglone is used as a substrate, the rate of a response is caused by the rate of enzymatic reactions (the metabolism of juglone in the presence of enzymes of a culture-receptor) and the rate of juglone transport into immobilized microbial cells. If CuSO_4_ inhibited only a monooxygenase–juglone interaction without affecting the rate of juglone transport into immobilized cells of *Rhodococcus* sp. 3, this explains the recorded nonsignificant inhibition of the response to juglone for immobilized cells compared to intact cells.

### 3.3. The Effect of Reactive Oxygen Species (ROS) on The Formation of The Response to Juglone for Intact Cells

In aerobic conditions, juglone is a H_2_O_2_-generating compound [29,30]. Obviously, ROS (H_2_O_2_) production occurs under the effect of the quinone form of juglone on microorganisms. Catalase, glutathione reductase, superoxide dismutase, peroxidase, and other enzymes take part in protecting microbial cells against oxidative stress.

Seemingly, ROS formed under the action of the quinone form of juglone could take part in the formation of the response to juglone for microbial cells. With the aim of checking this supposition and estimating susceptibility of the cells of the culture-receptor to ROS, cell responses to the quinone form or the phenolate form of juglone were measured in the presence of catalase. Since catalase degrades hydrogen peroxide, this enzyme should have inactivated H_2_O_2_ and protected cells against the harmful effects of this compound. All the strains of rhodococci (*Rhodococcus* sp. 3 is one of the rhodococci) under study, unlike pseudomonades, were catalase-positive (catalase activity was determined in the cells). Pseudomonades require externally introduced catalase to protect them from the action of H_2_O_2_. Thus, besides intact cells of *Rhodococcus* sp. 3, the cells of *Pseudomonas* sp. 4(c4) were applied for the formation of the laboratory model of the microbial reaction sensor.

**Figure 5:**
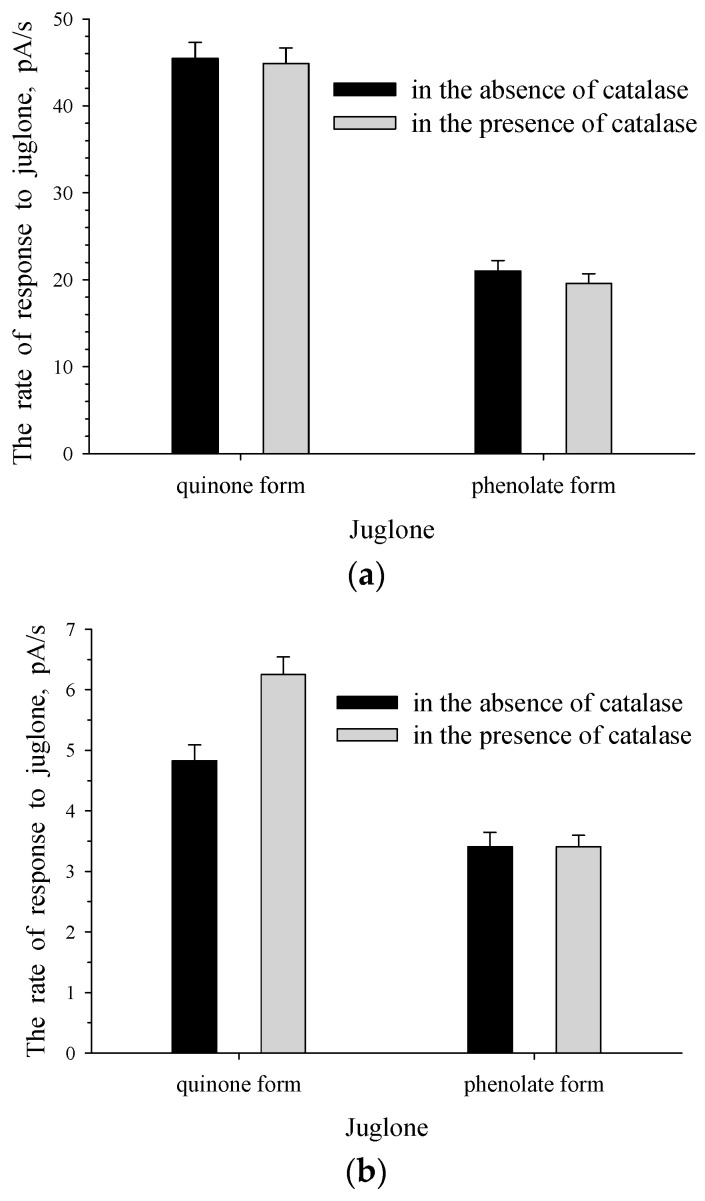
Response to juglone for intact cells of *Rhodococcus* sp. 3 (**a**) and *Pseudomonas* sp. 4(c4) (**b**) in the absence and presence of catalase.

Figure 5 presents the recorded responses of the intact cells of bacteria. It is clear that *Rhodococcus* sp. 3 and *Pseudomonas* sp. 4 (c4) display different levels of resistance to oxidative stress. H_2_O_2_ formed in the presence of the quinone form of juglone probably had an inhibitory effect on the cells of pseudomonade, leading to a decrease in the intensity of the cells’ response to juglone. In the presence of catalase, the effect of H_2_O_2_ on *Pseudomonas* sp. 4(c4) was inactivated, thereby confirming the findings of Zhang et al. [31] that catalase decreased the juglone-induced cell killing. In our research in the presence of extracellular catalase, H_2_O_2_ should not have a killing effect on intact cells of *Pseudomonas* sp. 4(c4), and juglone uptake into the cells should not have an inhibitory effect on the culture that can lead to an increase in the culture’s response to the substrate. This is what we observed. The increase in the response to the quinone form of juglone for *Pseudomonas* sp. 4(c4) intact cells was registered when the quinone form of juglone was used in the presence of catalase (Figure 5b).

As expected, H_2_O_2_ was formed only under the action of the quinone form, but not the phenolate form of juglone. Consequently, in the presence of catalase no changes were recorded in response to the phenolate form of juglone for intact *Pseudomonas* sp. 4 (c4) (Figure 5b). Unlike *Rhodococcus* sp. 3, *Pseudomonas* sp. 4 (c4) cells did not contain their own catalase and could not inactivate H_2_O_2_ themselves.

Intact cells of *Rhodococcus* sp. 3 (Figure 5a), unlike cells of *Pseudomonas* sp. 4(c4) (Figure 5b), did not respond to the catalase addition into the cell suspension. Catalase-positive cells of *Rhodococcus* sp. 3 possessed their own (intracellular) catalase, which protected them against ROS, without the need for the extra addition of extracellular catalase.

### 3.4. The Effect of ROS on The Formation of The Response to Juglone for Immobilized Cells

**Figure 6:**
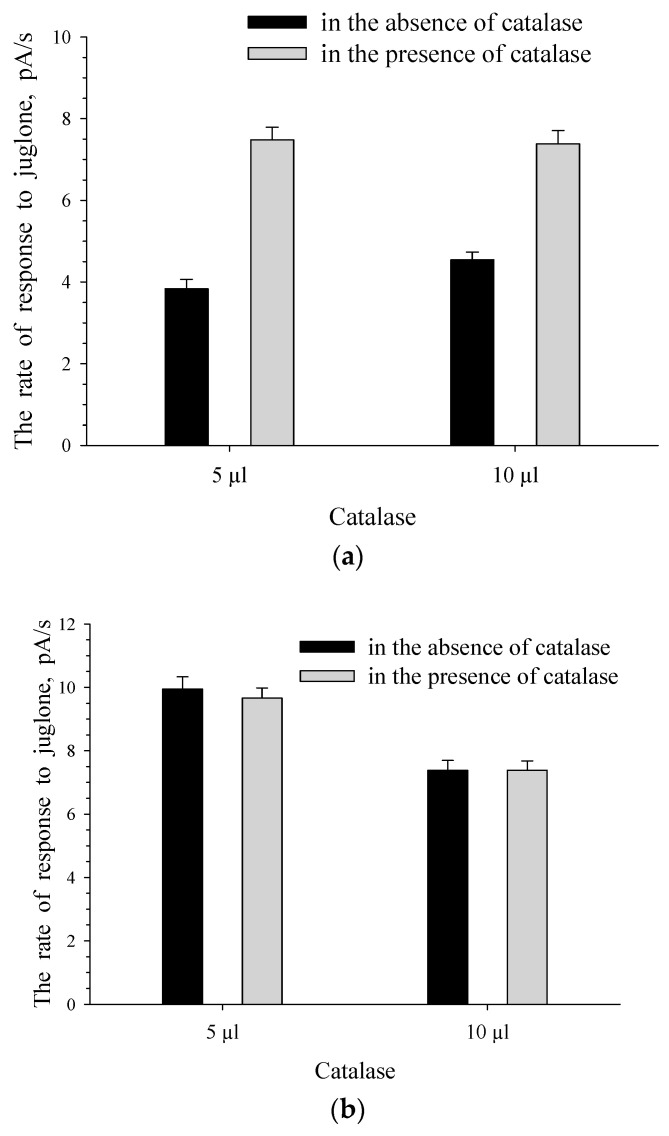
Response to the quinone form (**a**) and phenolate form (**b**) of juglone for immobilized cells of *Pseudomonas* sp. 4(c4) in the absence and presence of catalase.

Figure 6 shows that, in the presence of catalase, the activity of *Pseudomonas* sp. 4(c4) immobilized cells was similar to that of intact cells. As expected, no changes in the rate of the response to alkaline solution of juglone was observed for immobilized cells of *Pseudomonas* sp. 4(c4) (Figure 6b) similar to intact cells (Figure 5b) in the presence of catalase, since ROS is not formed in the presence of the phenolate form of juglone. For immobilized cells of the microbial sensor, it is known [26] that the response to the substrate is caused by the processes of substrate metabolism and substrate transport into the cells. Cells of *Pseudomonas* sp. 4 (c4) contained an enzyme that initiates the metabolism of juglone. This was evidenced by the presence of the response to juglone for intact culture cells (Figure 5b, the quinone form of juglone in the absence of catalase). The response to the quinone form of juglone was recorded for immobilized *Pseudomonas* sp. 4 (c4). Therefore, enzymatic transformation of the substrate took place in these cells. The process of metabolism of the substrate in cells could occur only after substrate transport into the immobilized bacterial cells. The presence of the response to the quinone form of juglone indicated the activity of both processes: the process of metabolism of juglone in cells and the process of transport of juglone into cells. When ROS formed in the presence of the quinone form of juglone was inactivated by the addition of catalase, the transport of juglone into the immobilized cells of the culture receptor did not inhibit the immobilized cells and the transport could be activated. Furthermore, an increase in the rate of substrate transport into the cells of the culture–receptor led to an increase in the rate of enzymatic conversion of substrate. Therefore, the addition of catalase to immobilized cells of *Pseudomonas* sp. 4(c4) was accompanied by activation of both processes, which caused the response of immobilized cells of the microbial membrane sensor—namely, substrate transport into the cells of the culture–receptor and enzyme–substrate interaction. If ROS influenced both these processes, then ROS had an effect on the formation of the response to the quinone form of juglone in immobilized bacterial cells. Thus, the response to the quinone form of juglone for immobilized cells of *Pseudomonas* sp. 4(c4) was caused by processes such as juglone transport into the cells of the culture–receptor, the inhibitory effect of juglone-induced ROS, and juglone metabolism catalyzed by cell’s enzymes.

No changes in the cell response to the quinone form of juglone were observed for immobilized cells of *Rhodococcus sp.* 3 in the presence of catalase. ROS had no effect on the formation of the response of catalase-positive *Rhodococcus sp.* 3 immobilized cells.

## 4. Conclusions

Thus, the results of this study demonstrated that models of the microbial reactor sensor and the microbial membrane sensor formed on the basis of cells of various cultures can be used to explore the activity of an enzyme, which initiates the metabolism of juglone, the process of transport of juglone into cells of a microorganism, and the effect of ROS on microbial cells.

## Data Availability

Data is contained within the article.
